# Supplementary material for: Thioredoxin-interacting protein regulates haematopoietic stem cell ageing and rejuvenation by inhibiting p38 kinase activity
Source: Nat Commun. 2016 Dec 8;7:13674. doi: 10.1038/ncomms13674 (PMC5155146; doi:10.1038/ncomms13674)
Supplement: Supplementary Information — Supplementary Figures 1-8. [file ncomms13674-s1.pdf]

Supplementary Figure 1

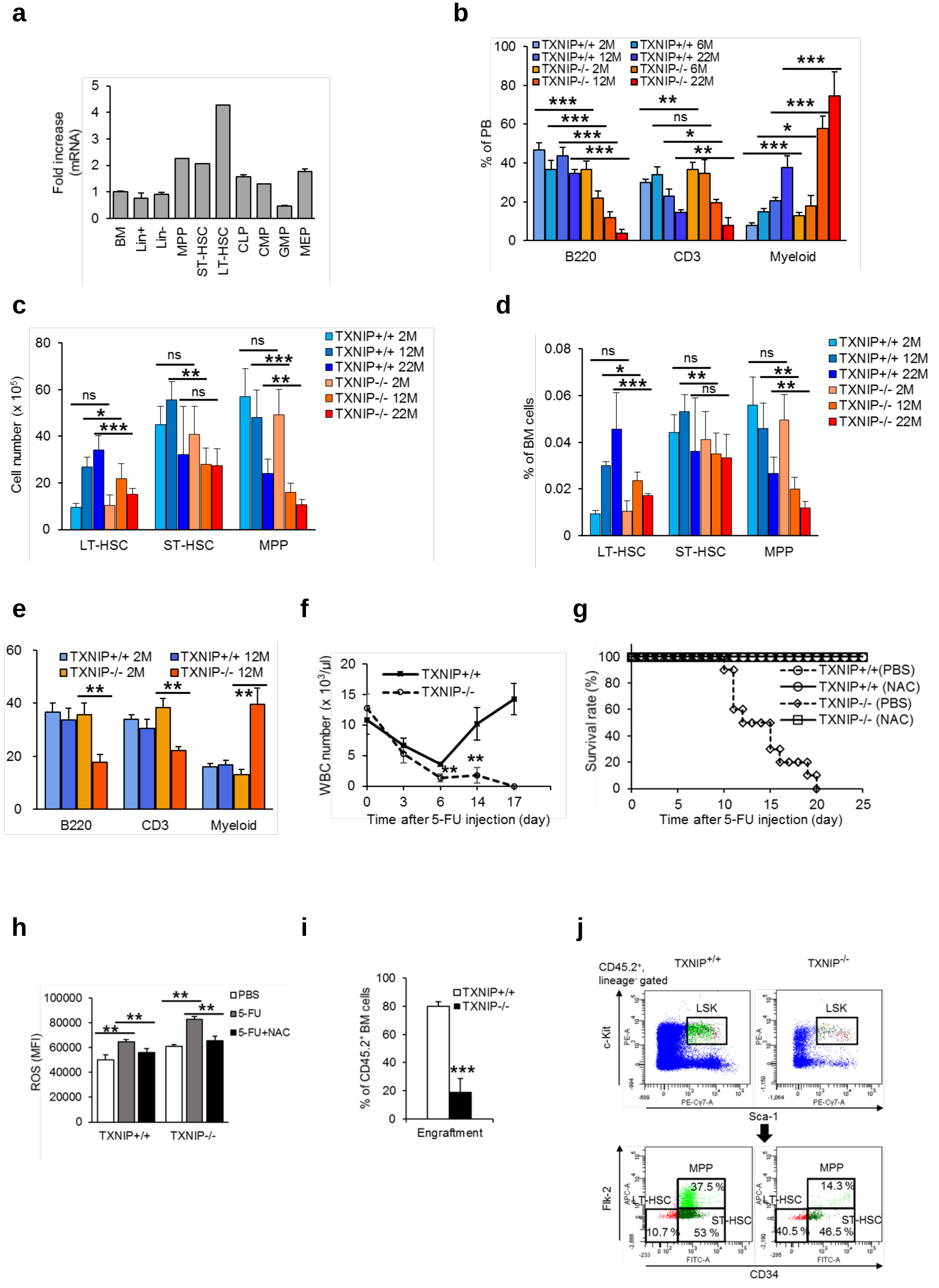

Supplementary Figure 1. Ageing phenotypes of *TXNIP*$^{-/-}$ HSCs in mice. (**a**) Quantitative real-time PCR of *TXNIP* in indicated subsets (n=3). (**b**) Percentage of B220^{+}, CD3^{+} and myeloid cells in PB. (**c,d**) Absolute number (**c**) and the frequency of LT-HSCs, ST-HSCs and MPPs in BM cells (**d**)(n=6). (**e**) Percentage of B220^{+}, CD3^{+} and myeloid cells in PB of female mice (n=7-10). (**f**) WBCs in PB after 5-FU administration (n=5-6). (**g**) Survival rate of young *TXNIP*$^{+/+}$ or *TXNIP*$^{-/-}$ mice after 5-FU (150 mg kg^{-1}) administration (n=10). NAC was i.p. injected 100 mg kg^{-1} daily starting at day -1. (**h**) ROS levels of LT-HSCs. We i.p. injected 5-FU (150 mg kg^{-1}) and NAC (100 mg kg^{-1}) then examined ROS in HSCs after 24 hours (n=3). (**i**) Percentage of donor-derived cells in BM (n=5 from 2 experiments). (**j**) A representative gating image of LT-HSCs, ST-HSCs and MPPs of LSKs among donor derived BM cells. Data are mean ± s.d. Statistical significance was determined using a two-tailed Student's *t*-tests. *P< 0.05, **P< 0.01, ***P<0.001.

Supplementary Figure 2

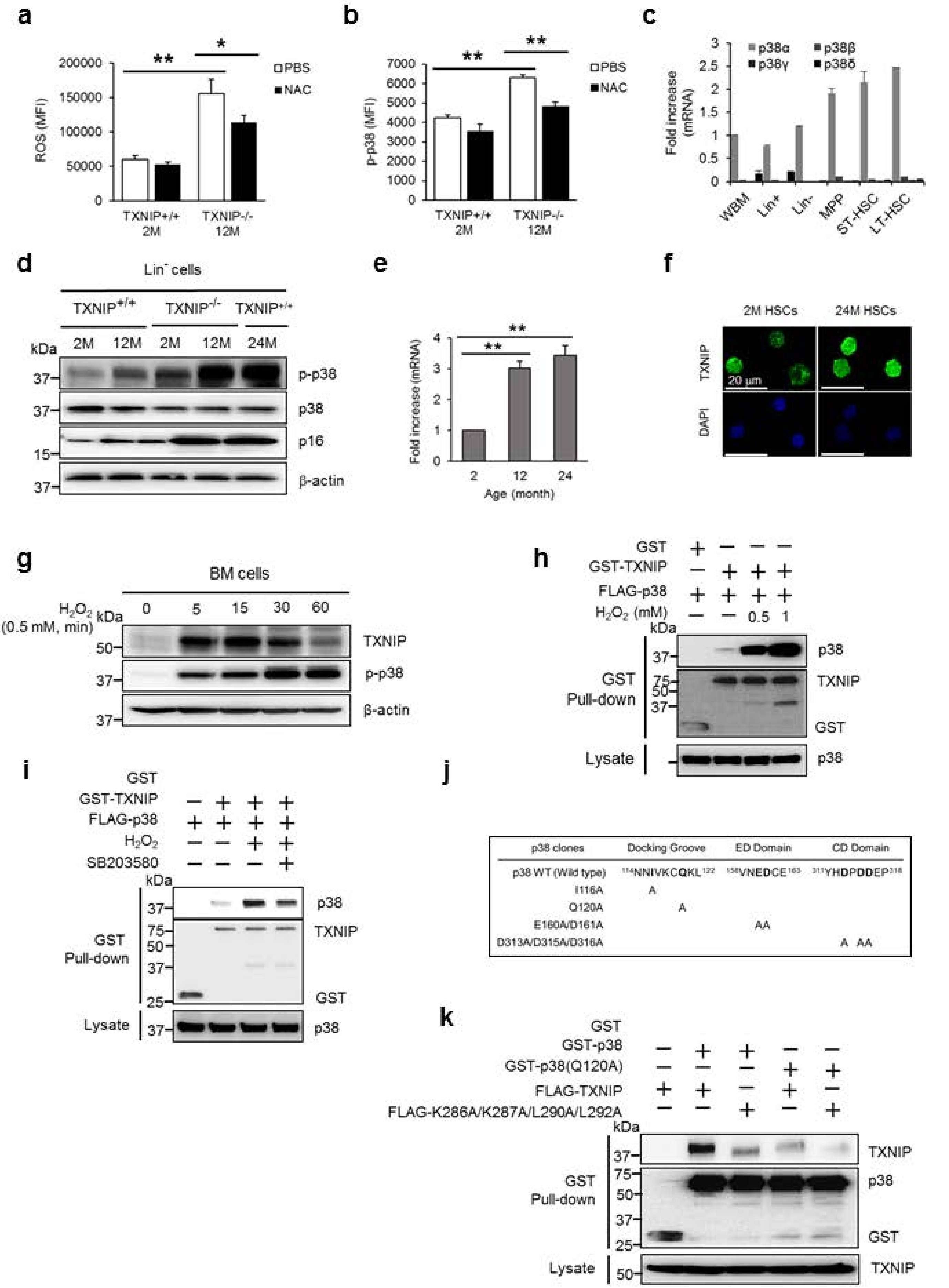

p38 clones	Docking Groove	ED Domain	CD Domain
p38 WT (Wild type)	^{114}NNIVKCQKL^{122}	^{158}VNEDCE^{163}	^{311}YHDPDDEP^{318}
I116A	A		
Q120A	A		
E160A/D161A		AA	
D313A/D315A/D316A			A AA

Supplementary Figure 2. Expression of p38 and TXNIP in HSCs and their interaction. **(a,b)** ROS (**a**) and phospho-p38 level (**b**) of LT-HSCs after NAC treatment. We i.p. injected 100 mg kg^{-1} NAC into mice then analyzed after 12 hours (n=3). (**c**) Quantitative real-time PCR of *p38* family in indicated subsets (n=3). (**d**) Western blot analyses of lin^- cells (repeated 3 times). (**e**) Real-time PCR of *TXNIP* in LT-HSCs (n=3). (**f**) Confocal images of TXNIP in LT-HSCs (repeated 3 times). (**g**) Western blot analyses of BM cells (repeated 2 times). (**h**,**i**) GST pull-down assay (repeated 3 times). (**j**) Constructed *p38* mutant clones. (**k**) GST pull-down assays between TXNIP and p38 mutants (repeated 3 times). Data are mean ± s.d. Statistical significance was determined using a two-tailed Student's *t*-tests. $^{**}P < 0.01$.

Supplementary Figure 3

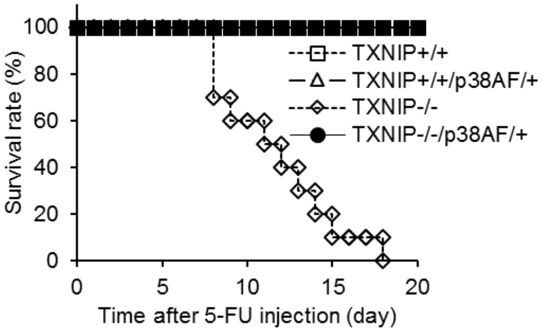

Supplementary Figure 3. Mice survival under haematopoietic stress.

Survival rate of young *TXNIP*$^{+/+}$ (n=10), *TXNIP*$^{-/-}$ (n=10), *TXNIP*$^{+/+}$/*p38*$^{AF/+}$ (n=7) or *TXNIP*$^{-/-}$/*p38*$^{AF/+}$ (n=9) mice after 5-FU (150 mg kg^{-1}) administration.

Supplementary Figure 4

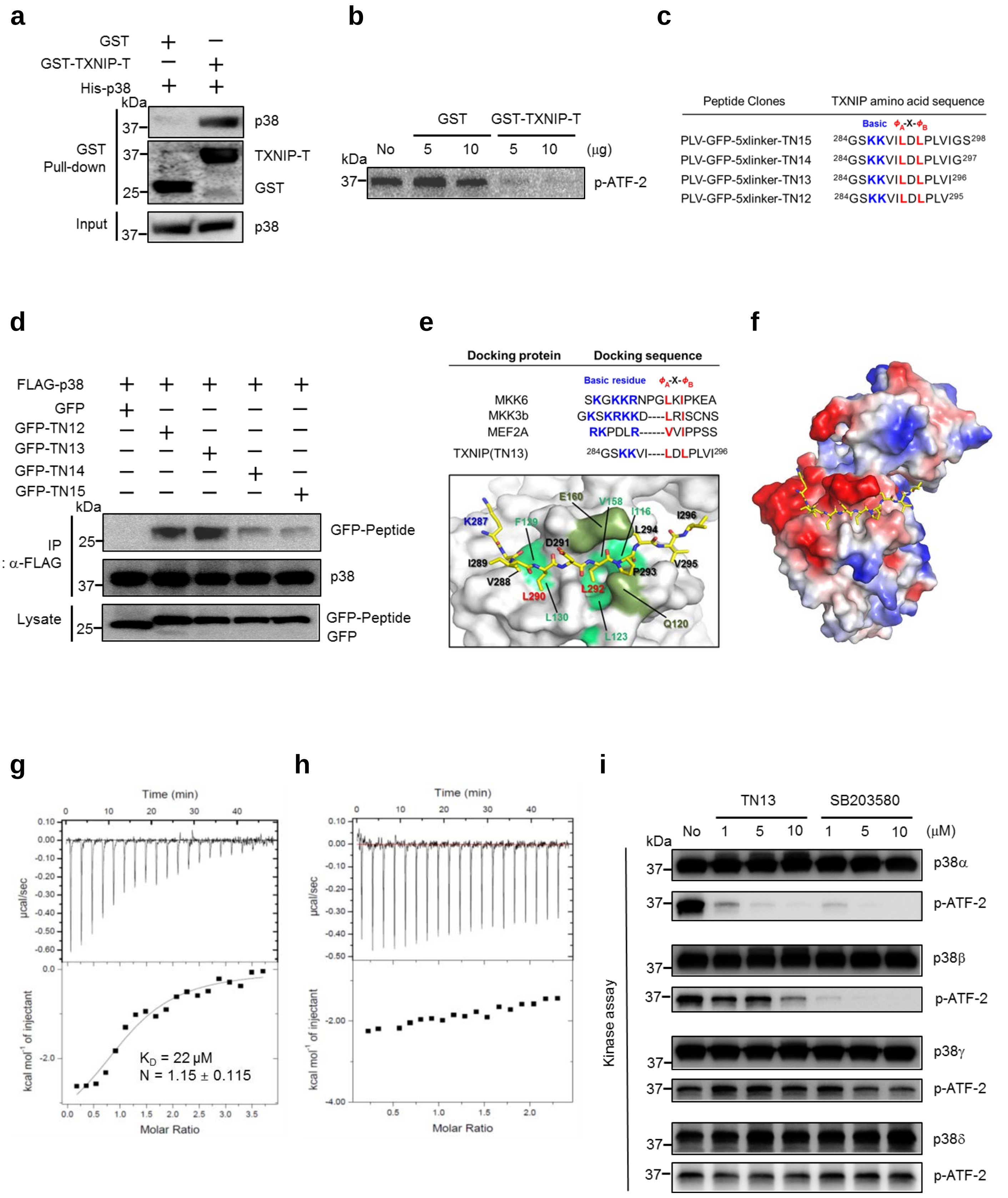

Supplementary Figure 4. TXNIP interacts with p38 and inhibits p38 kinase activity *in vitro*. (**a**) GST pull-down assay between His-p38 and GST or GST-TXNIP-T (repeated 3 times). (**b**) *In vitro* kinase assay for p38 (repeated 2 times). (**c**) Lenti-viral clones for *GFP*-peptide. (**d**) Immunoprecipitation assays for p38-GFP-peptide interaction (repeated 3 times). (**e**) Surface model of p38 with TXNIP peptide. (**f**) Surface model between p38 and TN13 peptide with electrostatic potentials. (**g**,**h**) Isothermal titration calorimetry (ITC) assay. His-p38 with TAT-TN13 (**g**) and His-p38 with TAT (**h**) (repeated 2 times). (**i**) *In vitro* kinase assay for p38 isoforms. (repeated 2 times).

Supplementary Figure 5

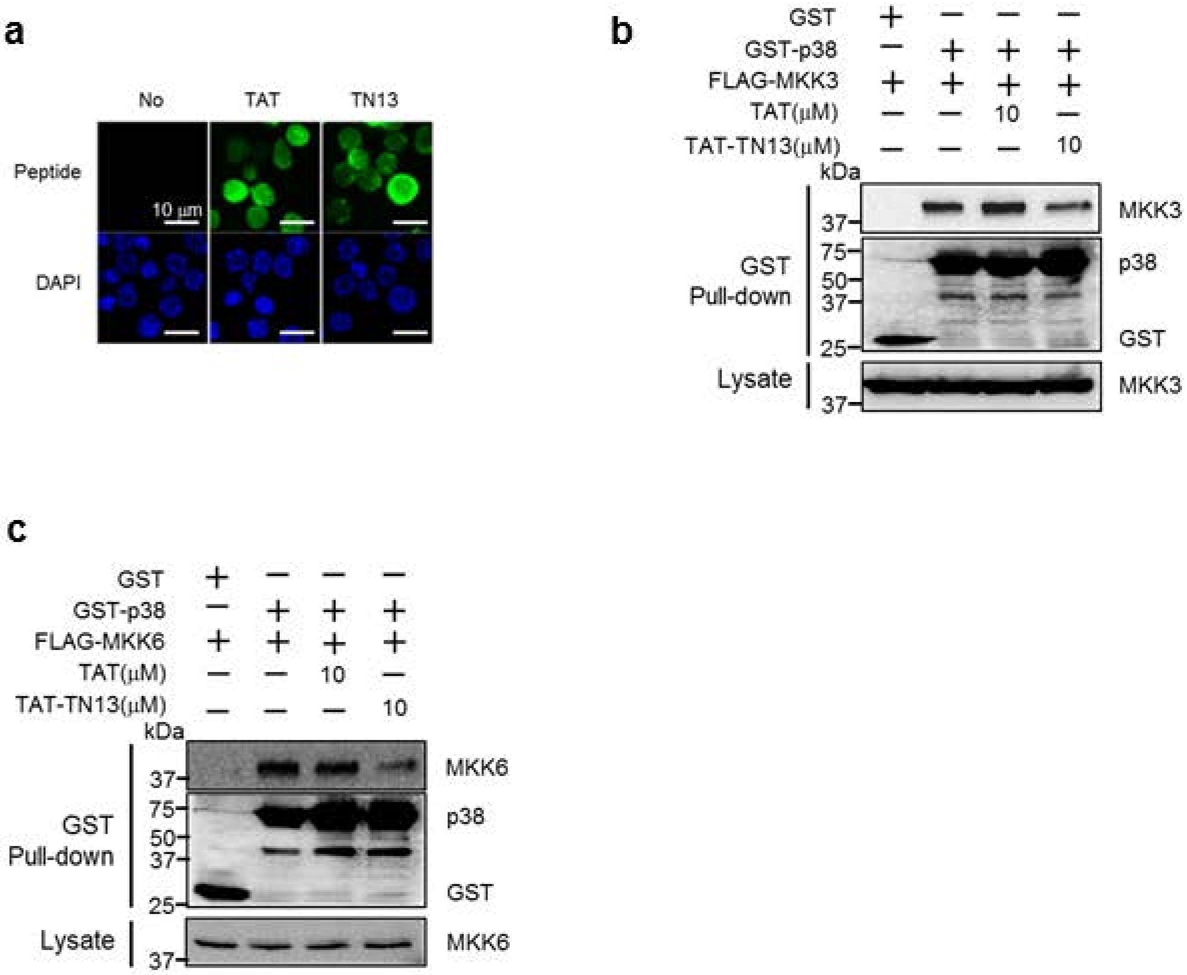

Supplementary Figure 5. TAT-TN13 peptide competes with MKK3 or MKK6 for p38 inhibition. (**a**) Confocal images of penetrated peptides in old HSCs. Sorted old HSCs were treated with 10 μM of TAT or TAT-TN13 in HSC media for 1 hour (repeated 2 times). (**b**,**c**) GST pull-down assay between p38 and MKK3 (**b**) or MKK6 (**c**) in 293T cells. Cells were treated with 10 μM of TAT or TAT-TN13 for 1 hour (repeated 2 times).

Supplementary Figure 6

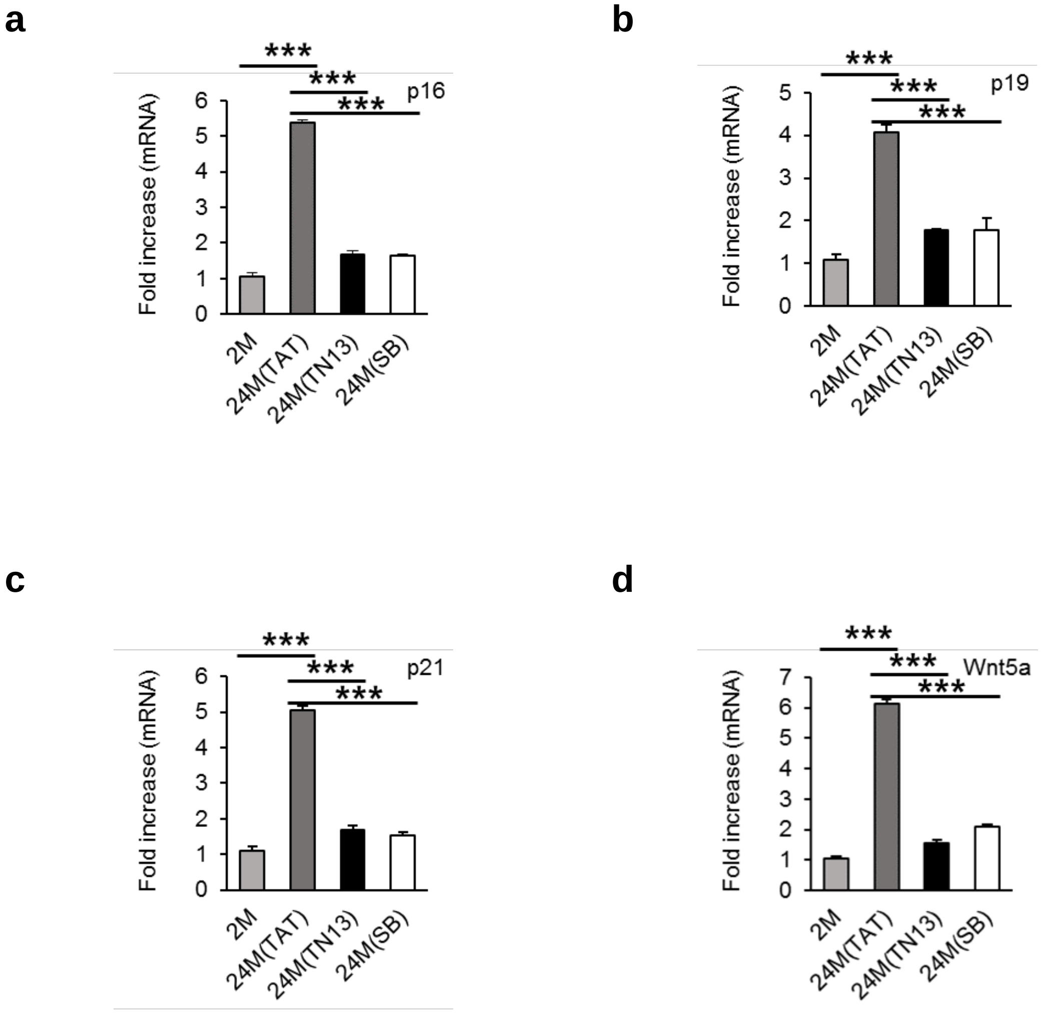

Supplementary Figure 6. Long-term regulation of ageing-associated genes in HSCs by TAT-TN13 treatment *in vivo*.

(a-d) Quantitative real-time PCR of ageing-associated genes in donor derived *CD45.2*$^{+}$ HSCs. After competitive transplantation assay, we isolated *CD45.2*$^{+}$ LT-HSCs from recipients and analyzed the expression of ageing associated genes using real-time PCR.

Supplementary Figure 7

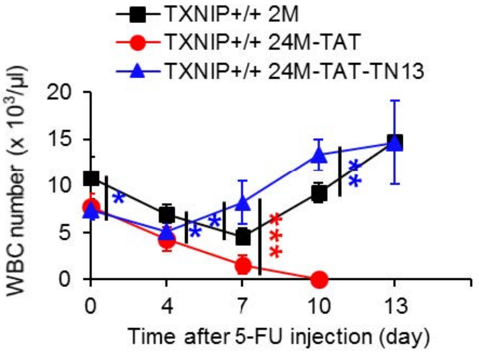

Supplementary Figure 7. Enhanced restoration of WBCs in old mice by TAT-TN13 treatment under haematopoietic stress.

WBCs in PB after 5-FU (100 mg kg^{-1}) administration (n=5-6 from 2 experiments). Data are mean ± s.d. Statistical significance was determined using a two-tailed Student's *t*-tests. *P< 0.05, **P< 0.01, ***P<0.001.

Supplementary Figure 8

Fig. 2e

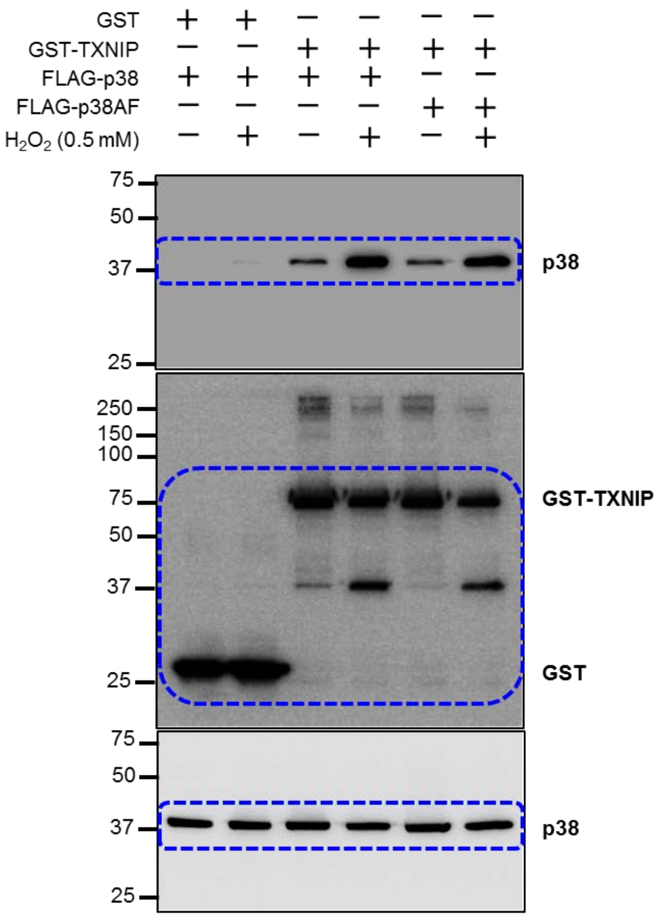

Fig. 2c

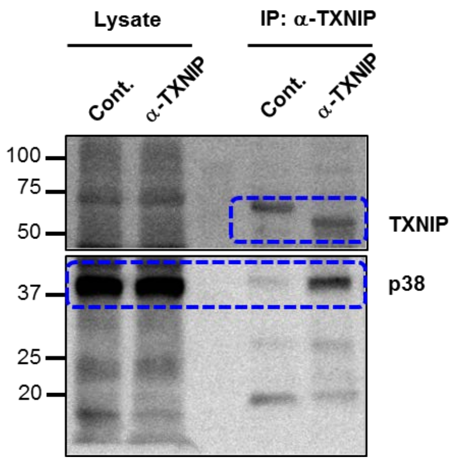

Fig. 2f

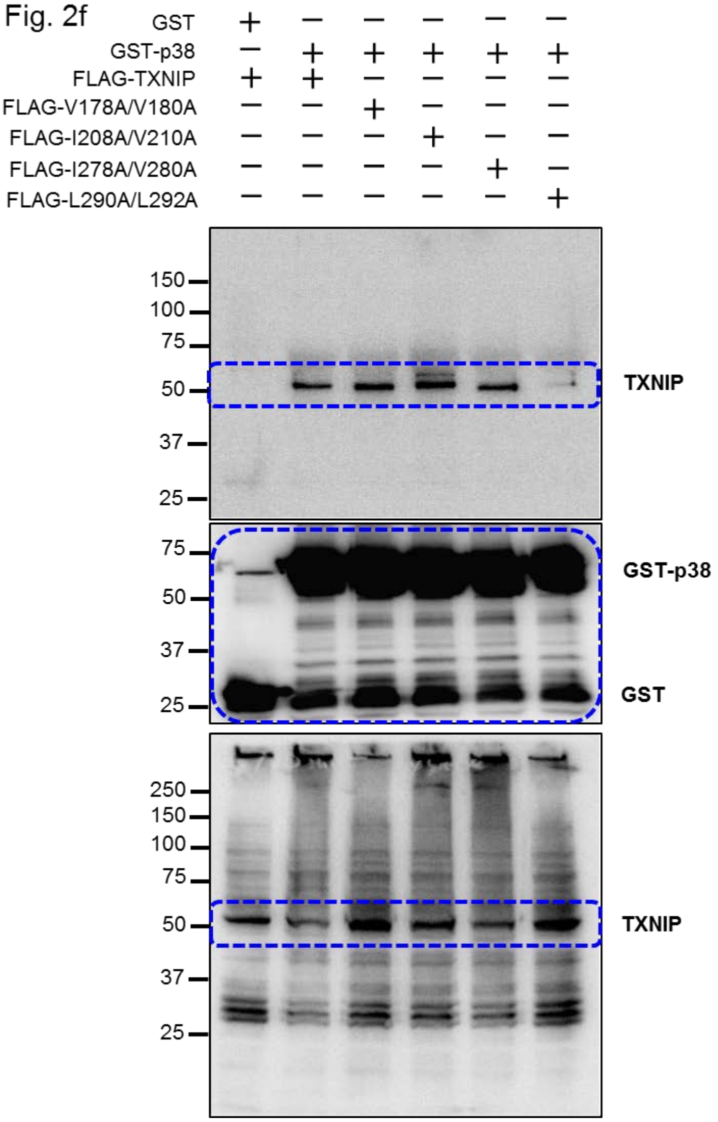

Supplementary Figure 8 Continued

Fig. 2g

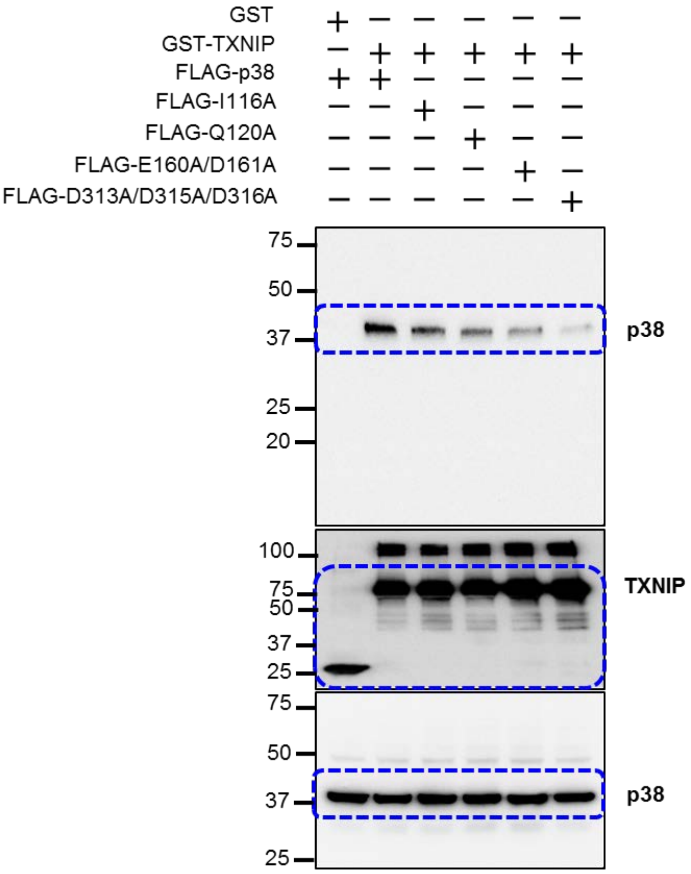

Fig. 4a

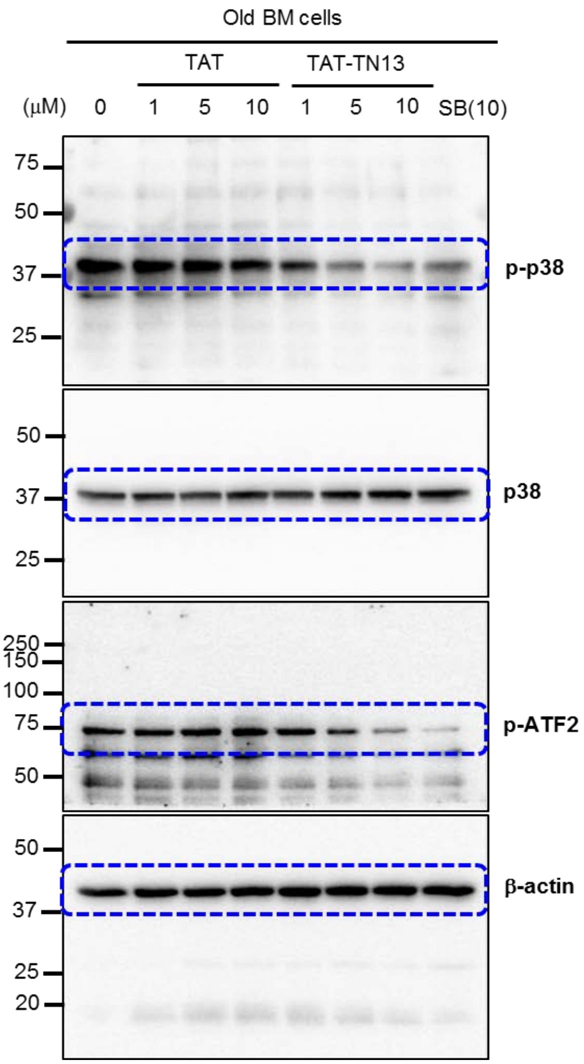

Supplementary Figure 8 Continued

Supplementary Fig. 2d

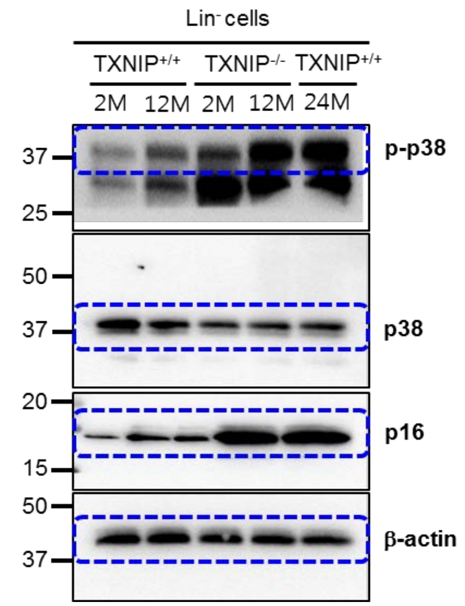

Supplementary Fig. 2h

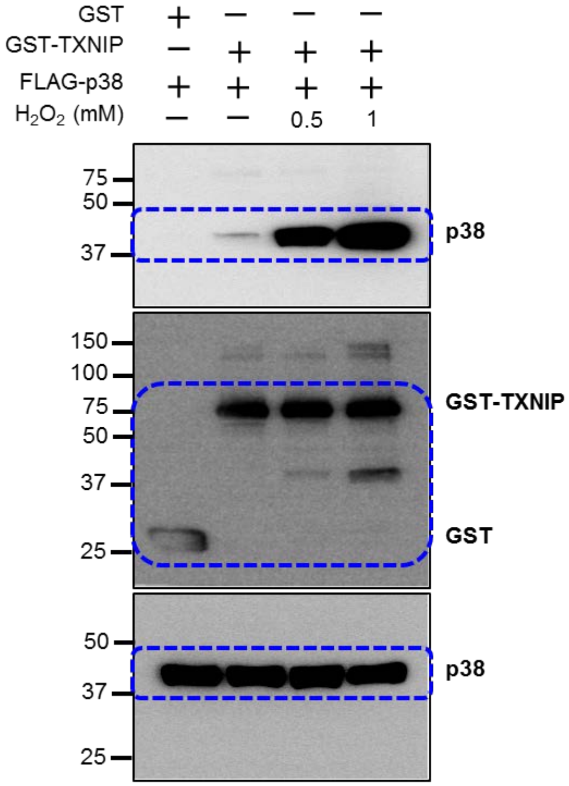

Supplementary Fig. 2k

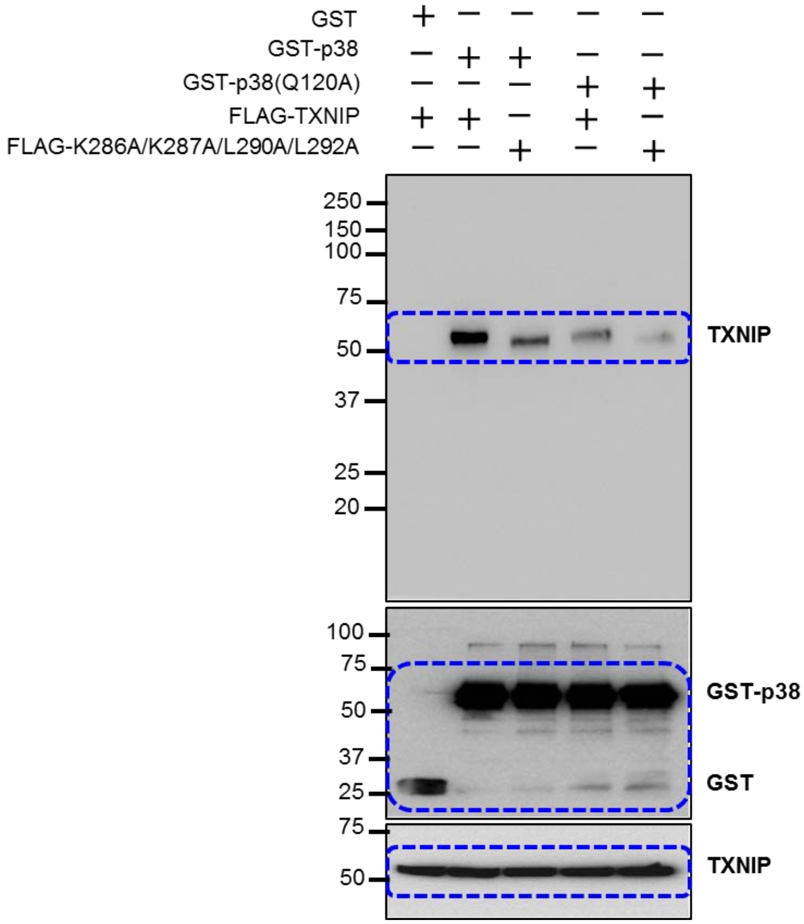

Supplementary Figure 8 Continued

Supplementary Fig. 4a

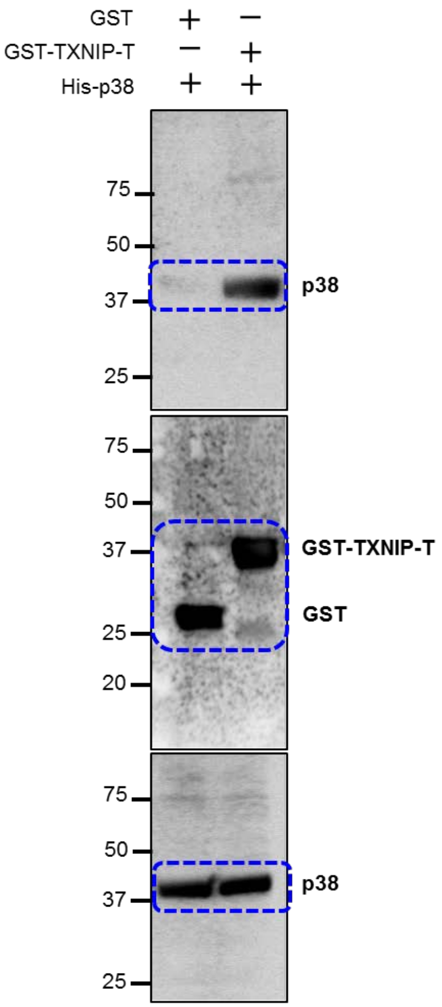

Supplementary Fig. 4d

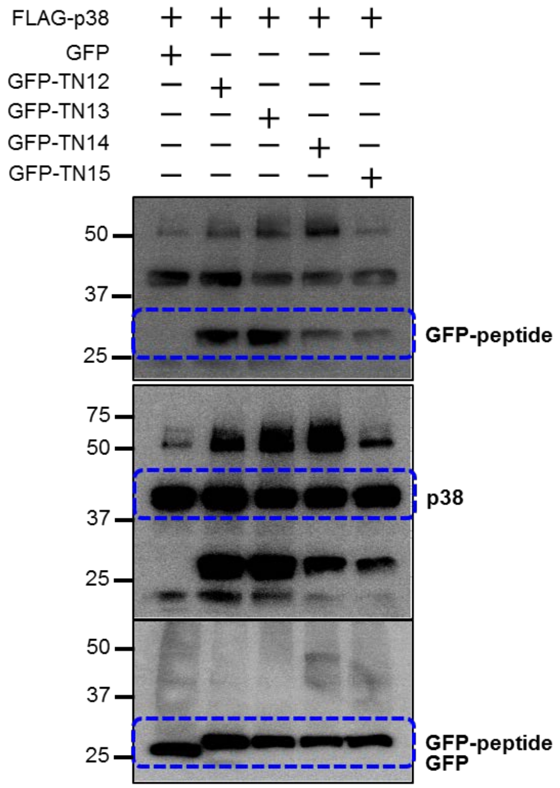

Supplementary Fig. 4i

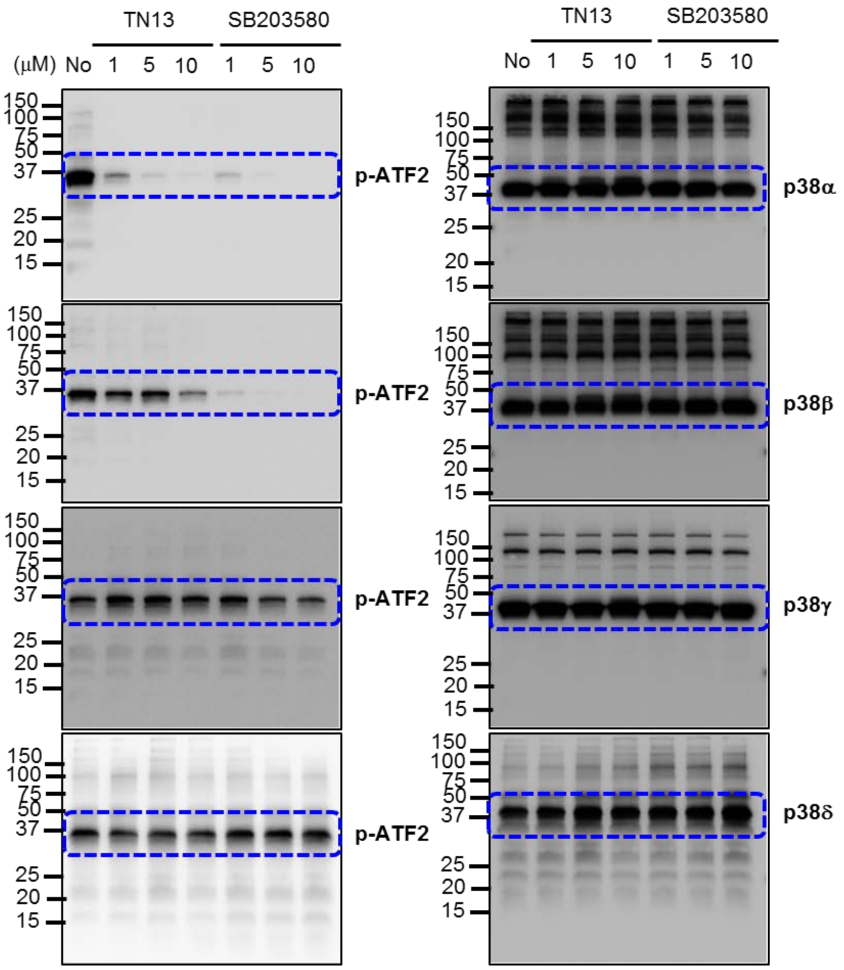

Supplementary Figure 8 Continued

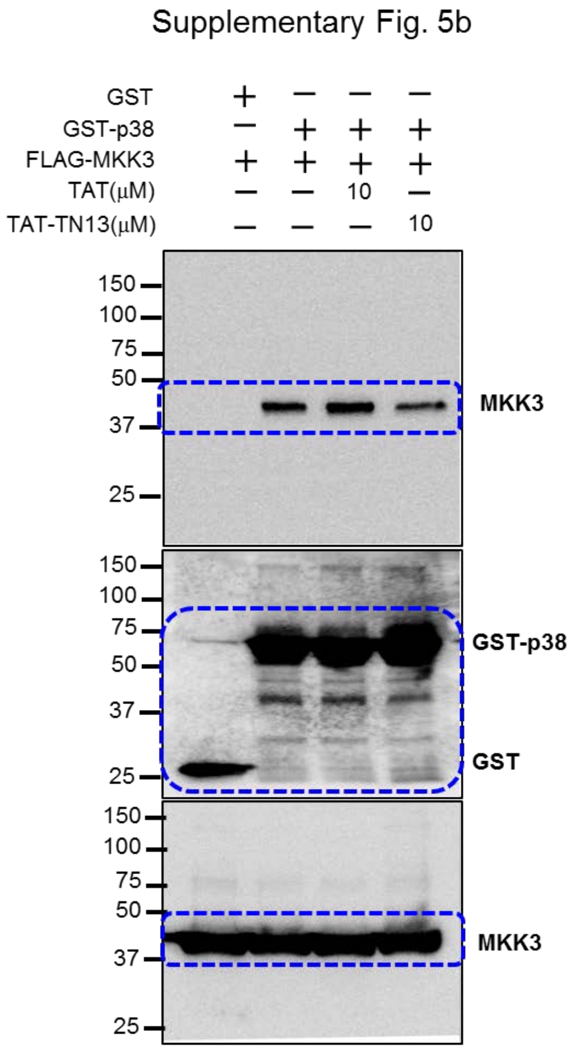

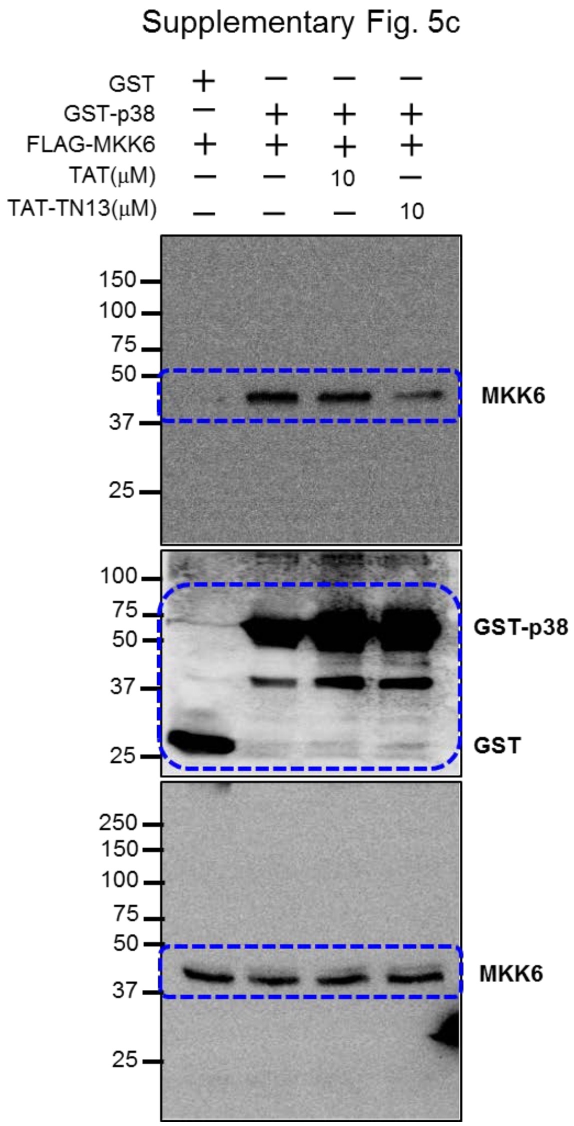

Supplementary Figure 8. Uncropped, full Western blot images of important blots in indicated figures (Fig. 2c, 2e, 2f, 2g, 4a, Supplementary Fig. 2d, 2h, 2k, 4a, 4d, 4i, 5b, 5c).
